# A Rare Presentation of Extramedullary Hematopoiesis as an Adrenal Mass: A Case Report

**DOI:** 10.7759/cureus.54598

**Published:** 2024-02-21

**Authors:** Sparsh Gupta, Anand Zingade, Mayur Baviskar, Kajari S Ashtaputre

**Affiliations:** 1 General Surgery, PCMC’S PGI Yashwantrao Chavan Memorial Hospital, Pimpri, Pune, IND

**Keywords:** total splenectomy, adrenalectomy, fibrohematopoetic tumor, sickle cell trait, retroperitoneal adrenal mass, extra-medullary hematopoiesis

## Abstract

Hematopoiesis is an enormous and complex process. When the primary site of hematopoiesis fails to meet the requirements of the body in conditions like hemoglobinopathies or myelofibrosis, various extramedullary sites take on the role of blood formation. Extramedullary hematopoiesis most commonly occurs in the liver, spleen, and lymph nodes and is rarely found in the thymus, heart, breast, adrenal glands, paravertebral regions, intraspinal tissue, and brain. Extramedullary hematopoiesis can mimic neoplasms in which symptoms are caused by the mass effect of the lesion. We report a rare case of a 41-year-old female patient with a fibrohematopoietic adrenal mass mimicking a neoplasm for which she underwent an adrenalectomy.

## Introduction

Hematopoiesis is an enormous and complex process. A model eliciting graded developmental progression of primitive, multipotent hematopoietic stem cells where they eventually lose one or more developmental potentials and finally become dedicated to a single cell lineage, which matures into the corresponding blood cell types was developed by hematologists [1]. When the primary site of hematopoiesis fails, as in hemoglobinopathies or myelofibrosis, various extramedullary sites take on the role of blood formation. Extramedullary hematopoiesis can be widespread, with the most common sites being the liver, spleen, and lymph nodes. In rare cases, extramedullary hematopoiesis can occur in the thymus, heart, breast, adrenal glands, paravertebral regions, intraspinal tissue, and brain. Extramedullary hematopoiesis can mimic neoplasms, in which symptoms are caused by the mass effect of the lesion [2-3]. We report the case of a 41-year-old female who underwent an adrenalectomy for an adrenal mass.

This article was previously presented as a poster at the 2023 ASICON, 83rd Annual Conference of The Association of Surgeons of India, in Visakhapatnam on 13th December 2023.

## Case presentation

**Figure 1:**
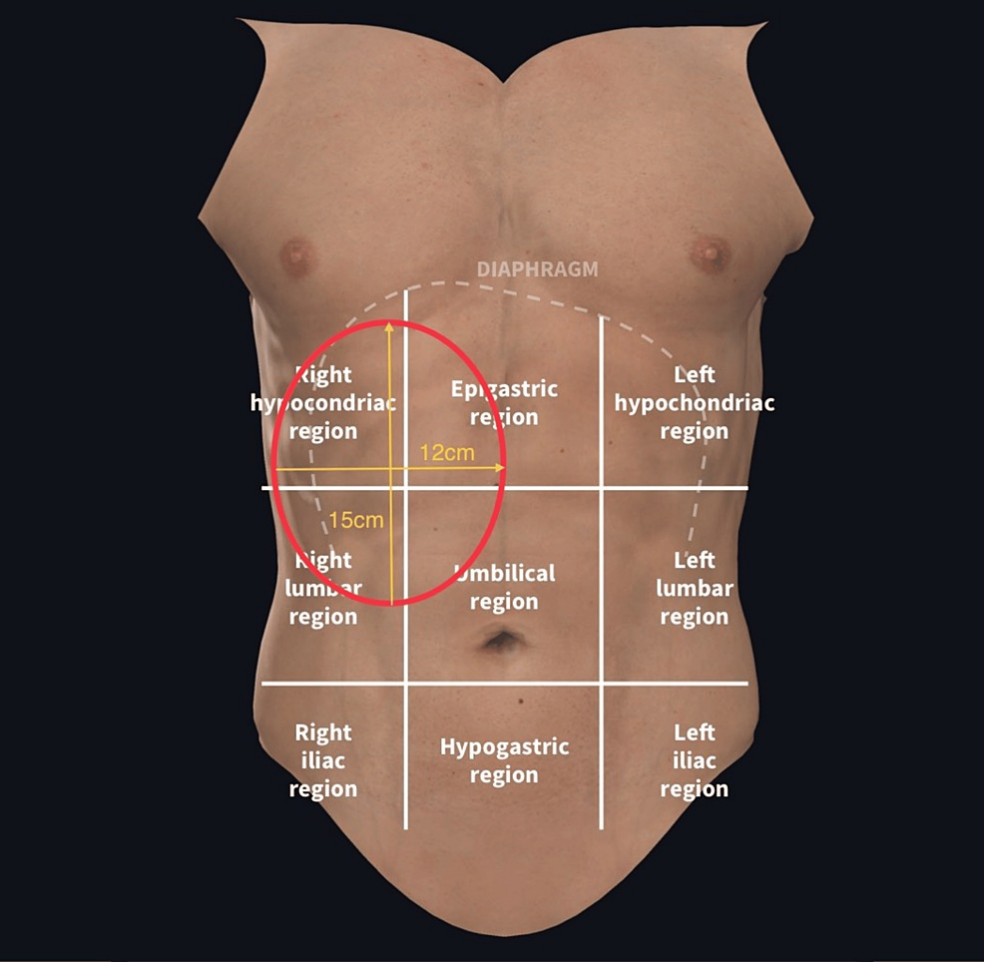
Abdominal segments showing the right suprarenal lump

A 41-year-old female presented with symptoms of upper abdominal pain for two years, which was more pronounced in the right hypochondrium and associated with early satiety and generalized weakness. The patient had a history of prior admissions for similar complaints, for which she had been transfused packed cell volume of around eight units one year ago, as well as another episode two years ago. On examination, the patient had abdominal fullness in the right hypochondriac regions, epigastric, right lumbar, and umbilical regions (Figure 1). On palpation, grade 3 splenomegaly was felt, along with an enlarged liver in the right hypochondriac region. There was a palpable mass of 15 x 12 cm in the right lumbar, hypochondriac, epigastric, and umbilical regions, which moved with respiration. There was also renal angle fullness on the right side. On cardiac auscultation, a pan-systolic murmur was heard.

**Figure 2:**
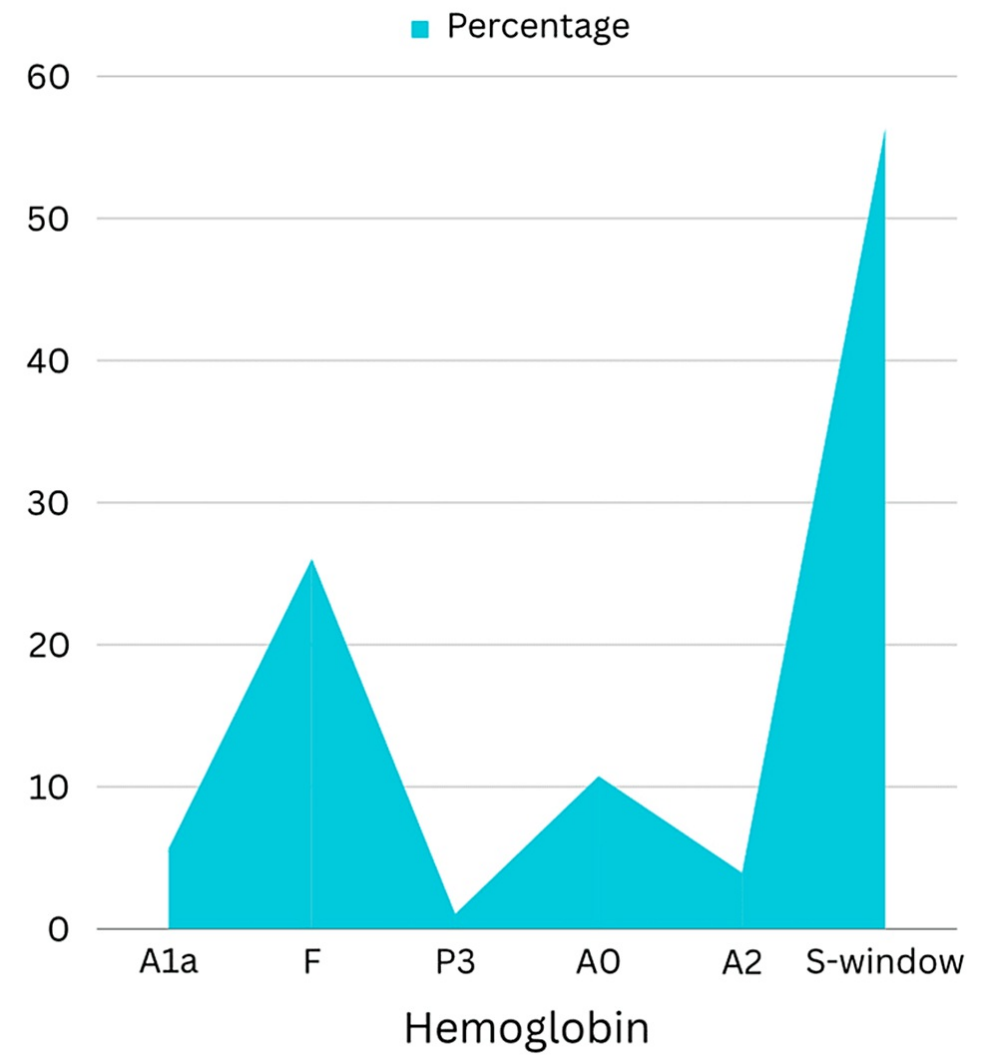
Area percentage chart depicting hemoglobin electrophoresis

Hematological investigations revealed the following findings at the time of admission: Hb: 1.5 gm%, platelets: 7740, retic count: 0.5%, and S. LDH: 3200 U/L. A peripheral smear showed pencil cells, tear drop cells, sickle cells, and fragmented RBCs, and a subsequent bone marrow aspirate revealed erythroid hyperplasia and suppressed myeloid series suggestive of reactive bone marrow. A sickling test was performed, which was positive, and a subsequent HPLC was suggestive of sickle cell trait (Figure 2). Urinary VMA and S. metanephrine were 9.86 mg/24 hours and 24.2 pg/ml, respectively.

**Figure 3:**
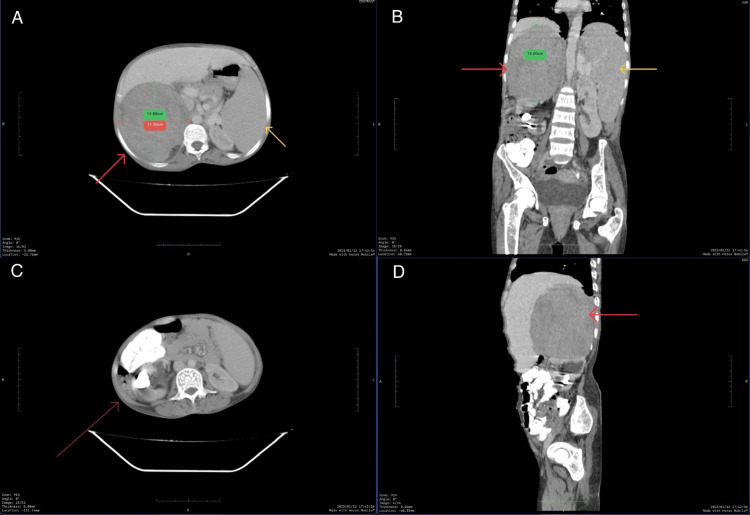
CT scan of abdomen and pelvis with contrast The images show (a) the axial section of the right suprarenal mass (red arrow) and splenomegaly (yellow arrow); (b) the coronal section of the right suprarenal mass (red arrow) and splenomegaly (yellow arrow); (c) axial section of right renal calculus (red arrow); and (d) sagittal section of right supra renal mass (red arrow) CT: computed tomography

**Figure 4:**
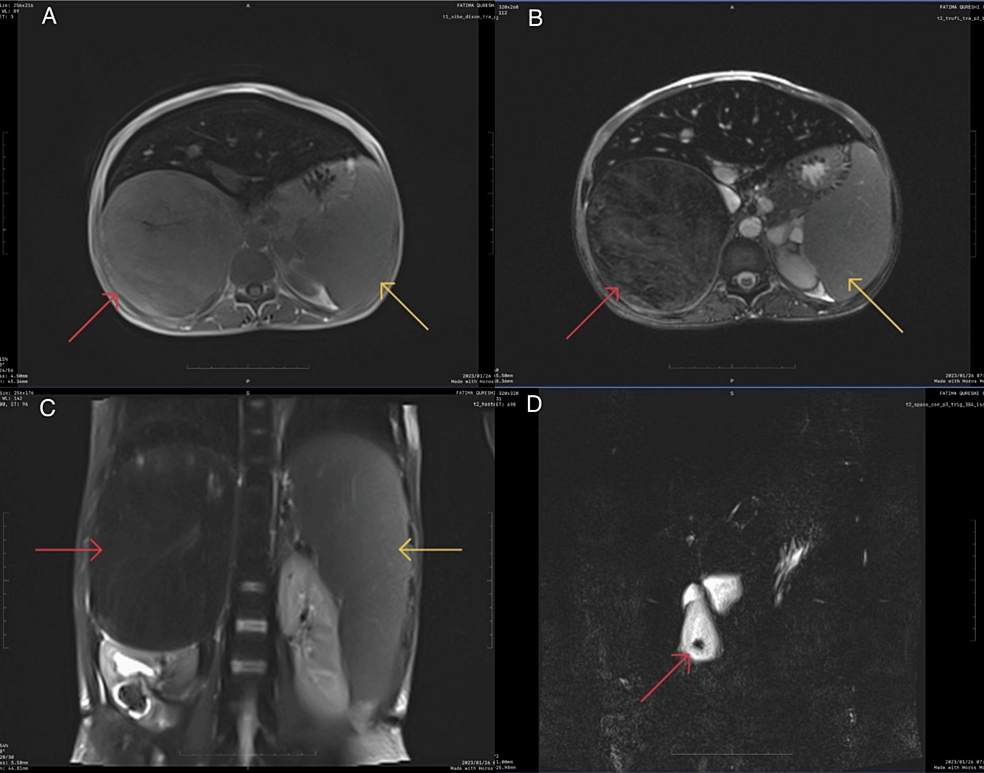
MRCP of the patient The images show (A) T1-weighted axial section of the right suprarenal mass (red arrow) with splenomegaly (yellow arrow); (B) T2-weighted axial section of the right suprarenal mass (red arrow) with splenomegaly (yellow arrow); (C) T2-weighted coronal section of the right suprarenal mass (red arrow) and splenomegaly (yellow arrow); and (D) T2-weighted coronal section with gall bladder calculus (red arrow) MRCP: magnetic resonance cholangiopancreatography

An ultrasonography was performed, which showed a right suprarenal mass with splenomegaly, a 1.2 cm calculus in the gall bladder, and a 2.4 cm calculus in the right renal pelvis. On further radiological investigations with CECT A+P (Figure 3) and MRCP (Figure 4), the findings of ultrasonography were confirmed, along with the presence of a well-defined 15 x 11.8 x 11.3 cm lobulated lesion with heterogenous post-contrast enhancement with non-enhancing necrotic areas in the right suprarenal region, displacing the kidney inferiorly, liver anteriorly, and portal vein and IVC medially without any vascular invasion. The left suprarenal gland appeared to be normal in shape and size.

**Figure 5:**
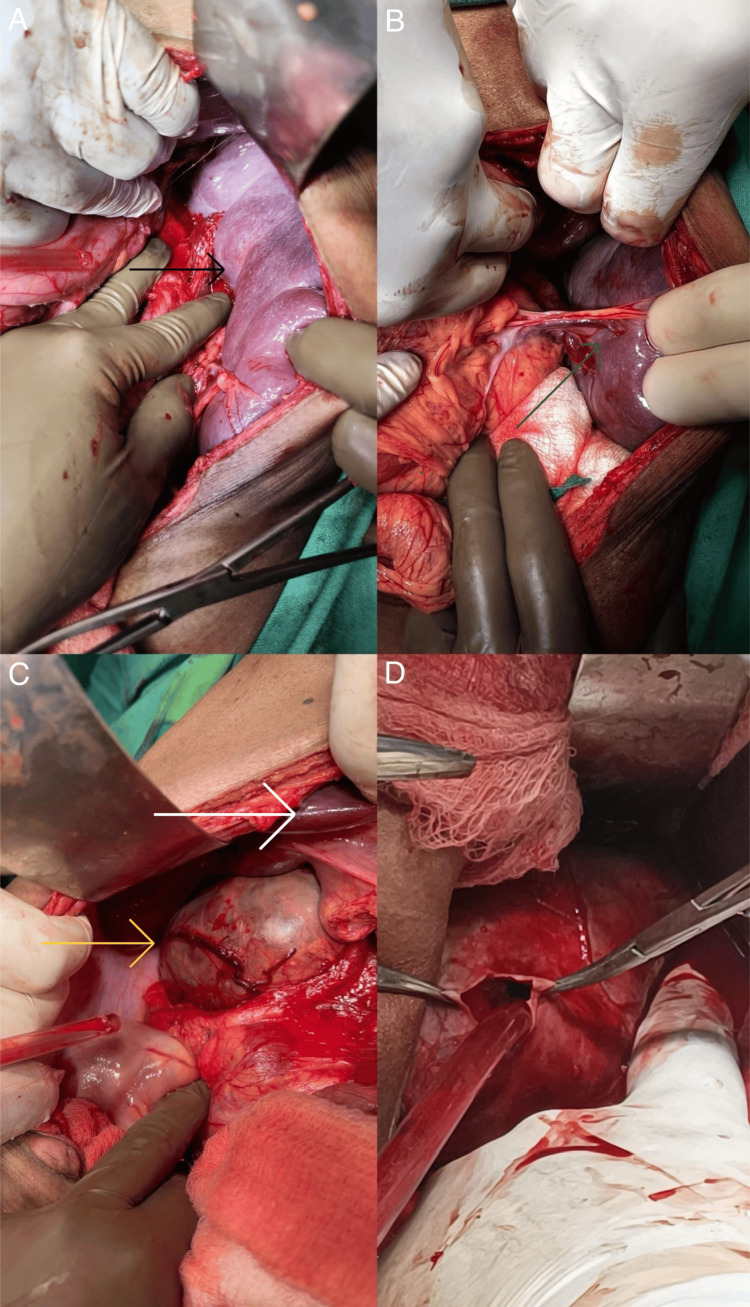
Intraoperative findings The images show (A) an enlarged spleen (black arrow); (B) a splenic hilum with vessels (green arrow); (C) the right suprarenal mass (yellow arrow) under the liver (white arrow); and (D) suctioning from the semi-cystic mass

An elective operative procedure was planned for splenectomy and the removal of suprarenal mass after adequate optimization of the patient. Intraoperatively (Figure 5), an enlarged spleen measuring 21 cm, and a 15 x 12 x 12 right suprarenal mass abutting the IVC, with multiple feeding vessels from the IVC, were observed. There was a 1 cm stone in the gall bladder. Intraoperative blood loss was around 2.0 L, mainly from feeder vessels from the IVC. Multiple transfusions were required, and the patient was shifted to the critical care unit, intubated, and on ionotropic support.

**Figure 6:**
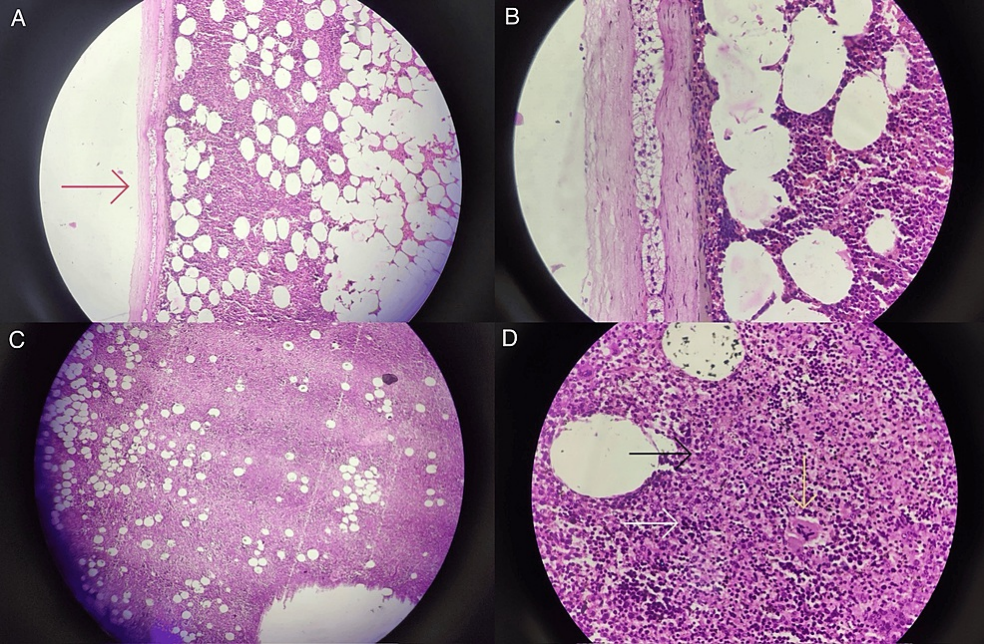
Histopathological images The images show (A) a low-power field of the adrenal gland compressed to the periphery (red arrow) and extramedullary hematopoiesis; (B) a high-power field showing the adrenal gland with extramedullary hematopoiesis; (C) extramedullary hematopoietic tissue with megakaryocytes; and (D) high-power field showing hematopoietic tissue with megakaryocytes (yellow arrow), erythroid precursors (white arrow), and myeloid precursors (black arrow)

Histopathology reports were suggestive of a 15 x 11.5 x 7.5 cm, 820.5 g, single, capsulated, well-circumscribed mass. On cut sections, mahogany brown, smooth, and shiny, with hemorrhagic areas, were seen. On microscopic examination, a compressed adrenal gland at the periphery with hematopoietic tissue consisting of numerous megakaryocytes, erythroid, and myeloid precursors suggestive of extramedullary hematopoiesis in the adrenal gland was observed (Figure 6). On immunohistochemistry testing, the hematopoietic cells showed positivity for CD3 (focal) and MP0 (focal) but were negative for CK, synaptophysin, S100, chromogranin, inhibin, CD34, and SMA.

## Discussion

The spleen functions by removing old and defective erythrocytes, thereby assuring quality control over red blood cells. It also plays an immunogenic role by synthesizing antibodies, along with destroying antibody-opsonized bacteria and blood cells. Splenomegaly may result from a rise in these daily functions of the spleen. The spleen also helps by assisting the host to adapt to a hostile environment. The destruction of bacteria and particulates, the production of an immune response to specific pathogens, and the creation of cellular components of blood when the marrow is unable to meet the demands of the body are examples of adaptive functions of the spleen (extramedullary hematopoiesis). Hypersplenism is a condition characterized by splenomegaly, cytopenias, and normal or hyperplastic bone marrow, and it is a response to splenectomy. Increased cellular element destruction as a result of decreased blood flow via larger and congested cords (congestive splenomegaly) or immune-mediated mechanisms cause cytopenias. Increased reticulocyte production index should correspond with increased erythrocyte production in the marrow, albeit the value might be lower than anticipated due to increased reticulocyte sequestration in the spleen [4].

Sickle cell disease is a homozygosity for the sickle hemoglobin mutation, while the sickle cell trait is a heterozygous mutation. Sickle erythrocytes irreversibly polymerize when deoxygenated, and their membranes could be permanently damaged. Sickle erythrocytes lead to clinical and laboratory phenotypes of disease. One of the complications involves an acute anemic episode where there is excess splenic sequestration, a delayed hemolytic transfusion reaction, with the destruction of transfused and sometimes autologous red cells. This is treated by RBC transfusion and splenectomy if there are more than one or two episodes of sequestration. Another complication includes gallstones, which occur in 40% of patients and, if symptomatic, can be treated with laparoscopic cholecystectomy [5].

Adrenocortical carcinoma is a rare neoplasm with a bimodal age distribution, with increased incidence in children and individuals in their fourth to fifth decade of life. Approximately 50% of adrenocortical tumors are non-functioning. The most common manifestations of a non-functioning tumor are an expanding abdominal mass and back or abdominal pain. The sensitivity, specificity, and likelihood ratio of tumor size in predicting malignancy were reported to be 96%, 51%, and 2 for tumors >4 cm, and 90%, 78%, and 4.1 for tumors >6 cm [6].

Incidentalomas are adrenal tumors found during imaging procedures for unrelated purposes [7]. However, extramedullary hematopoiesis in the adrenal gland is uncommon [8]. The escape of progenitor cells from the marrow and their lodgement in other organs contribute to extramedullary blood cell formation. Some patients are asymptomatic at the time of diagnosis, while in symptomatic patients, fatigue, weakness, shortness of breath, pruritus, and palpitations are nonspecific but frequent complaints [9-13]. In research examining how patients respond to myelofibrosis treatment, the phrase "constitutional symptoms" is frequently used to describe the combined occurrence of fever, weight loss, and night sweats [14]. At the time of diagnosis, almost all patients have splenomegaly, and two-thirds of patients have detectable hepatomegaly [9-13]. Foci of hematopoiesis may become clinically apparent as fibrohematopoietic tumors in the adrenal glands [15-16], renal parenchyma, and lymph nodes.

## Conclusions

Due to stress on the body caused by the increased destruction of erythrocytes secondary to underlying sickle cell anemia, the body may show a propensity to recruit extramedullary sites for blood cell production to meet the elevated demands. Patients with hepatosplenomegaly or hematological disorders may present with an abdominal mass. This mass, in rare cases, is discovered to be arising from the adrenal gland. Extramedullary hematopoiesis presenting as a fibrohematopoietic tumor of the adrenal gland is a very rare occurrence. However, despite its rarity, it should be considered in the differentials for incidentaloma or adrenocortical carcinoma. In patients with hematological disorders presenting with an adrenal mass, an image-guided biopsy can be considered for further management.
